# A Comprehensive Study Monitoring the Venom Composition and the Effects of the Venom of the Rare Ethiopian Endemic Snake Species *Bitis parviocula*

**DOI:** 10.3390/toxins13050299

**Published:** 2021-04-22

**Authors:** Vladimír Petrilla, Magdaléna Polláková, Barbora Bekešová, Zuzana Andrejčáková, Radoslava Vlčková, Dana Marcinčáková, Monika Petrillová, Eva Petrovová, Drahomíra Sopková, Jaroslav Legáth

**Affiliations:** 1 Department of Biology and Physiology, The University of Veterinary Medicine and Pharmacy, Komenského 73, 041 81 Košice, Slovakia; vladimir.petrilla@uvlf.sk (V.P.); barbora.bekesova@student.uvlf.sk (B.B.); zuzana.andrejcakova@uvlf.sk (Z.A.); radoslava.vlckova@uvlf.sk (R.V.); drahomira.sopkova@uvlf.sk (D.S.); 2 Zoological Department, Zoological Garden Košice, Široká 31, 040 06 Košice-Kavečany, Slovakia; 3 Department of Pharmacology and Toxicology, The University of Veterinary Medicine and Pharmacy, Komenského 73, 041 81 Košice, Slovakia; dana.marcincakova@uvlf.sk (D.M.); jaroslav.legath@uvlf.sk (J.L.); 4 Department of General Competencies, The University of Veterinary Medicine and Pharmacy, Komenského 73, 041 81 Košice, Slovakia; monika.petrillova@uvlf.sk; 5 Department of Morphological Disciplines, The University of Veterinary Medicine and Pharmacy, Komenského 73, 041 81 Košice, Slovakia; eva.petrovova@uvlf.sk; 6 Department of Biotechnology and Bioinformatics, Faculty of Chemistry, Rzeszow University of Technology, Powstańców Warszawy 6, 35-959 Rzeszów, Poland

**Keywords:** *Bitis parviocula*, vasoactivity, haemotoxicity, acetylcholinesterase, cytotoxicity, chicken embryo

## Abstract

The Ethiopian endemic snake of the species *Bitis parviocula,* recognized for its colorful patterns, might be more interesting as we look deeper into the venom activity. We assayed the effects of venoms from the most widespread venomous African *Bitis arietens* and closely related species *Bitis parviocula* using The Hen’s Egg Test—Chorioallantoic membrane test (HET-CAM) and Chicken embryotoxicity screening test (CHEST), acetylcholinesterase (AChE) analysis, cytotoxicity assay performed on cell lines and protein analysis of selected venoms. Our results indicated that *B. parviocula* venom contains vasoactive compounds that have a direct effect on blood vessels. The AChE analysis showed significant ability inhibiting AChE activity in embryonic tissue. Cytotoxicity observed on A549 ATCC^®^ CCL-185™ cells indicates the possible presence of cytotoxic agents in *B. parviocula* venom. We proved previously described differences in the composition of venom obtained from *B. arietans* and *B. parviocula* by using electrophoresis and total protein concentration. Based on similarities in vasoactive effects observed after administration of venoms onto a chicken chorioallantoic membrane, we suggest that venom from *B. arietans* and *B. parviocula* might share certain venom proteins responsible for haemotoxicity. The main active components of *B. parviocula* venom are unknown. Our results suggest that it might be worth performing proteomic analysis of *B. parviocula* venom as it might contain medically valuable compounds.

## 1. Introduction

The Ethiopian endemic species of mountain adder *Bitis parviocula* of the family Viperidae is a rare and little-studied species of venomous snake. The name “parviocula” is composed of the Latin words “parvus”, which means small, and “oculus”, which means eye. *B. parviocula* snakes inhabit areas on both sides of the East African Rift, from the east in the Bale Mountains National Park and in the Bonga, Jimma and Bedele localities on the west side [1].

Until 1995, all knowledge concerning *B. parviocula* was obtained from the findings of only three individuals. The three specimens known until 1995 came from localities on both sides of the East African Rift Valley. Two snakes were found in forested areas on the west side. One of the snakes was caught in a forest clearing in the area of an old coffee plantation, while the other snake was found in a forest town, hiding in the grass of the brewery complex. The third known individual came from the eastern side and emerged in tall grassy areas near a rocky stream [2,3]. The *B. parviocula* species seems to be poorly understood, most likely due to the lack of valid information. Recent findings have revealed that museum specimen collected from Dodola in Ethiopia in the late 1960s, previously incorrectly considered an unusually patterned example of *B. parviocula*, is in fact a member of a different Ethiopian species of *Bitis harenna* [4].

Very little is known about *B. parviocula* snakes, especially about the venom composition, effects and mechanism of action. Only one experimental study was carried out comparing the composition and activity of *B. parviocula* venom and the closely related African puff adder, *Bitis arietans*. The authors explained that after the agreement in 2007, the species *B. parviocula* began to pave the way for enthusiasts of the snake trade. However, the placement of *B. parviocula* species into many private and zoological collections increases the likelihood of accidental envenomations, whereas emergency physicians are encouraged to treat venomous snakebites that have not been previously documented or described. According to suspected cross-reactivity with polyvalent antivenoms produced against other species of *Bitis*, researchers had decided to experimentally neutralize the *B. parviocula* venom with SAIMR (South African Institute of Medical Research) antivenom. Based on the results obtained, the authors suggest that the lethal activity of the venom from *B. parviocula* can be very effectively neutralized by SAIMR antivenom; therefore, SAIMR antivenom should be considered useful in the treatment of envenomations caused by these snakes [5].

The ability to degradate α and β chains of fibrinogen, the potent effect on platelets, and the haemorrhagic activity comparable to the venom of *Crotalus viridis* suggest that *B. parviocula* venom, which is traditionally thought to be cytotoxic [5], may contain haemotoxic compounds with vasoactive properties. It was proved that the chicken chorioallantoic membrane is a useful model for the visualization of snake-venom-induced vascular injury [6]. We decided to employ the model of chicken chorioallantoic membrane (CAM) and The Hen’s Egg Test—Chorioallantoic membrane test (HET-CAM) for visualization and evaluation of the effects of *B. parviocula* and *B. arietans* venom on vasculature. An embryotoxic assay that employs the model of chicken embryo and Chicken embryotoxicity screening test (CHEST) methodology was carried out using both venoms. To test neurotoxic activity of both venoms, the acetylcholinesterase (AChE) activity was analyzed in tissue samples.

Our research is in accordance with the Three Rs rules, which can be defined as an effort to increase animal welfare, to reduce and replace experimental animals, and to refine the way experiments are carried out to ensure minimal suffering. In chicken embryos, the pain receptivity does not fully develop until embryonic day 13. For the purposes of our study, the chicken embryos were dissected and processed on embryonic day 9, which means that the experimental part requiring the use of live embryos was performed before the embryos could experience painful stimulation [6,7].

The highest cytotoxic effects that venoms show is certainly a promising way to utilize crude venom or at least its individual components in the antitumor activity of the cells. In preliminary tests of clinical studies, the antitumor effects of the raw venom and their isolated enzymes were observed.

Cytotoxicity of snake venom toxins is associated with their specificity and affinity with cells and tissues. The specificity of the measures can be used in treatment of various diseases. Certain proteins or peptides derived from snake venoms are able to bind to the membranes of cancer cells, and this ability also affects the migration and proliferation of these cells. Several studies of venoms obtained from cobra species found that there are isolated cytotoxins that readily penetrate human lung adenocarcinoma cells (A549) and assemble in lysosomes [8].

The cellular response to toxins can be assessed by various assays. We used the xCELLigence system (RTCA), which evaluates intracellular changes (proliferation, adherence and morphology) in real time throughout the entire experiment. However, the system does not evaluate the actual state inside the cell so additional endpoint analyses, such as cell viability assessment (MTS test), need to be used.

The venom composition (*B. parviocula* and *B. arietans*) was analyzed using agarose gel electrophoretic analysis and determination of protein concentration, as other authors have already reported some differences in the number of protein bands present in *B. parviocula* and *B. arietans* venoms [5].

The employment of different methods highlights the aim of this study to present novel information, thereby broadening the spectrum of *B. parviocula* compared to the most widespread African viper, *B. arietans*.

## 2. Results

Samples of whole crude snake venoms obtained from *B. arietans* and *B. parviocula* were diluted using saline solution to concentration of E-1 (106 µg/µL), E-2 (10.6 µg/µL), E-3 (1.06 µg/µL), E-4 (0.106 µg/µL), and E-5 (0.0106 µg/µL) respectively.

### 2.1. Chicken Embryo Based Experimental Assays

#### 2.1.1. HET-CAM

**Figure 1:**
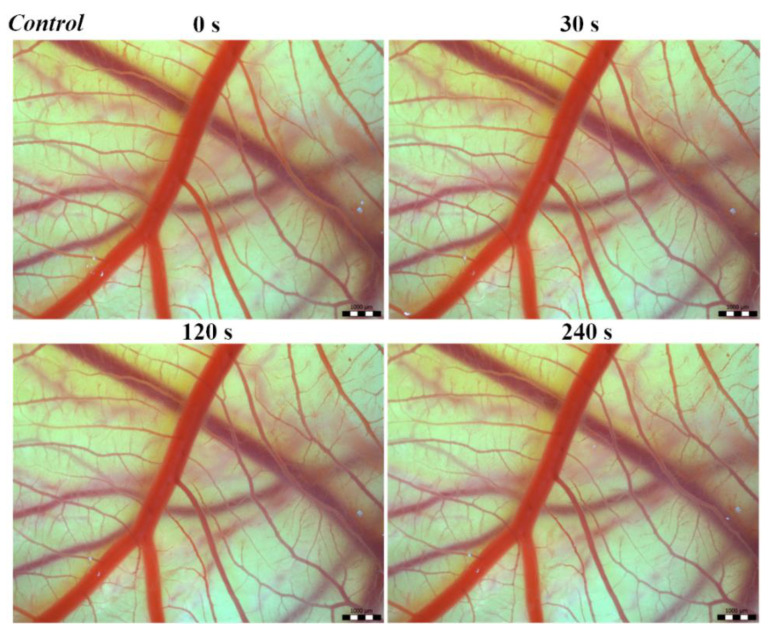
Photos of The Hen’s Egg Test—Chorioallantoic membrane (HET-CAM) test of the control group of chicken embryos, scale bar: 1 mm.

Vascular injury can be assessed using the vascular network of chicken CAM. After administration of both *B. arietans* and *B. parviocula* venoms, we observed local hyperaemia of individual vessels, followed by massive haemorrhage. Extensive haemorrhagic areas also considerably reduced the visibility of other effects. In a control group, sterile saline solution was used and no effects were observed. It confirms that the vascular injury was caused by the snake venoms (Figure 1).

**Figure 2:**
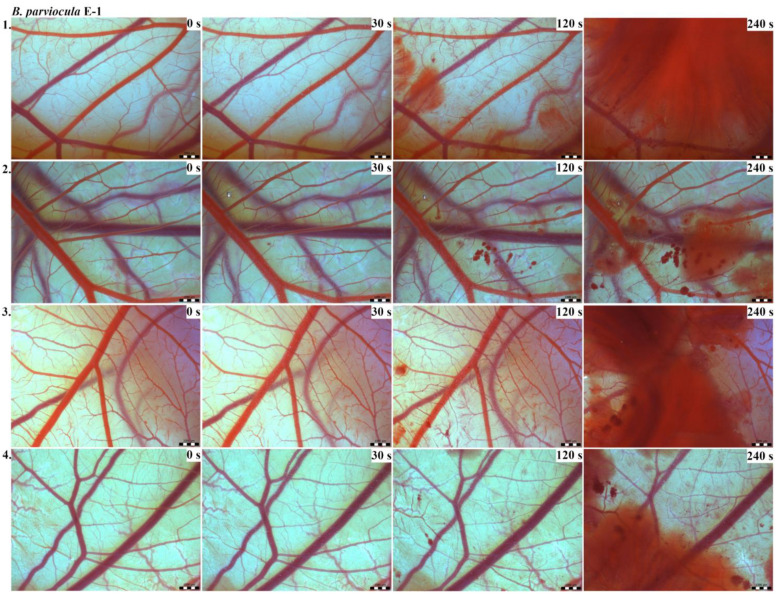
Photos of the HET-CAM test for *B. parviocula* venom, concentration E-1, scale bar: 1 mm.

**Figure 3:**
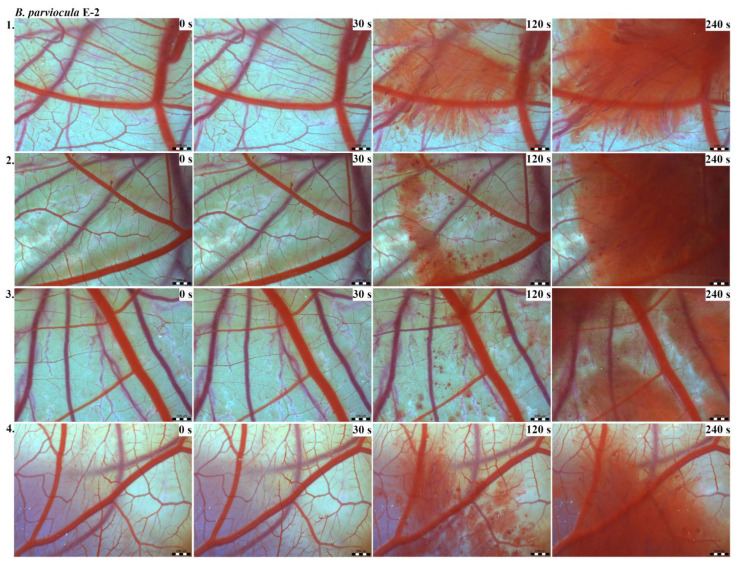
Photos of the HET-CAM test for *B. parviocula* venom, concentration E-2, scale bar: 1 mm.

**Figure 4:**
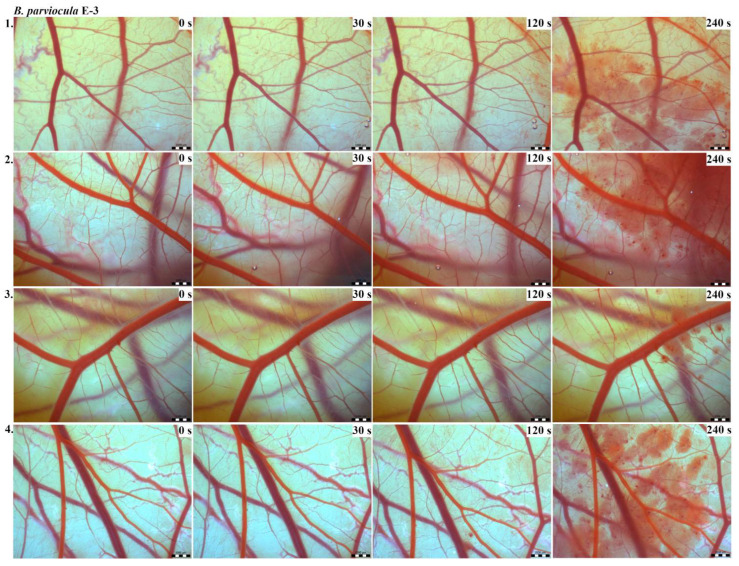
Photos of the HET-CAM test for *B. parviocula* venom, concentration E-3, scale bar: 1 mm.

**Figure 5:**
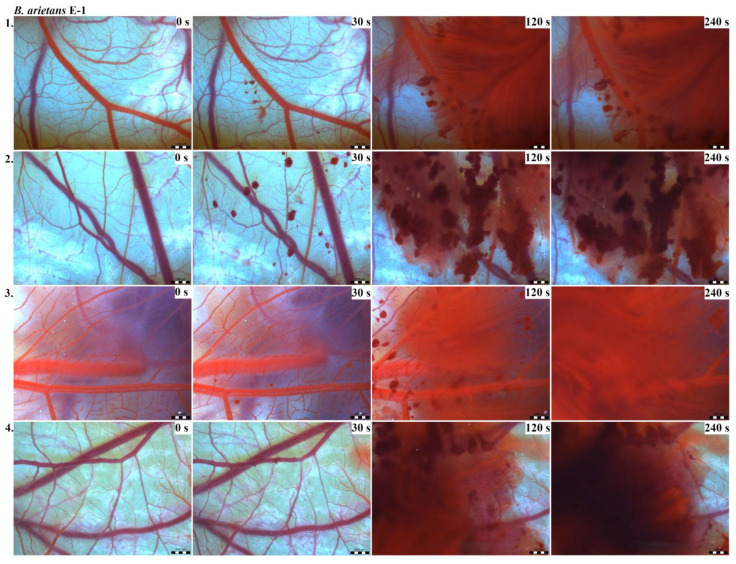
Photos of the HET-CAM test for *B. arietans* venom, concentration E-1, scale bar: 1 mm.

**Figure 6:**
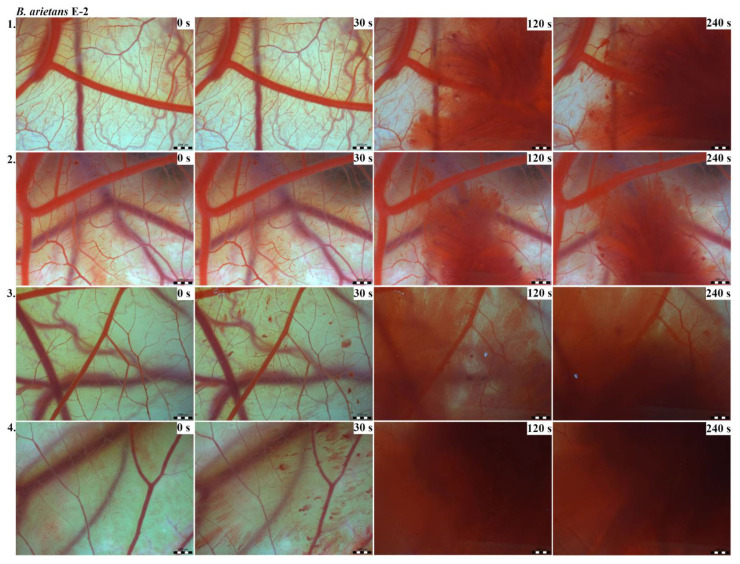
Photos of the HET-CAM test for *B. arietans* venom, concentration E-2, scale bar: 1 mm.

**Figure 7:**
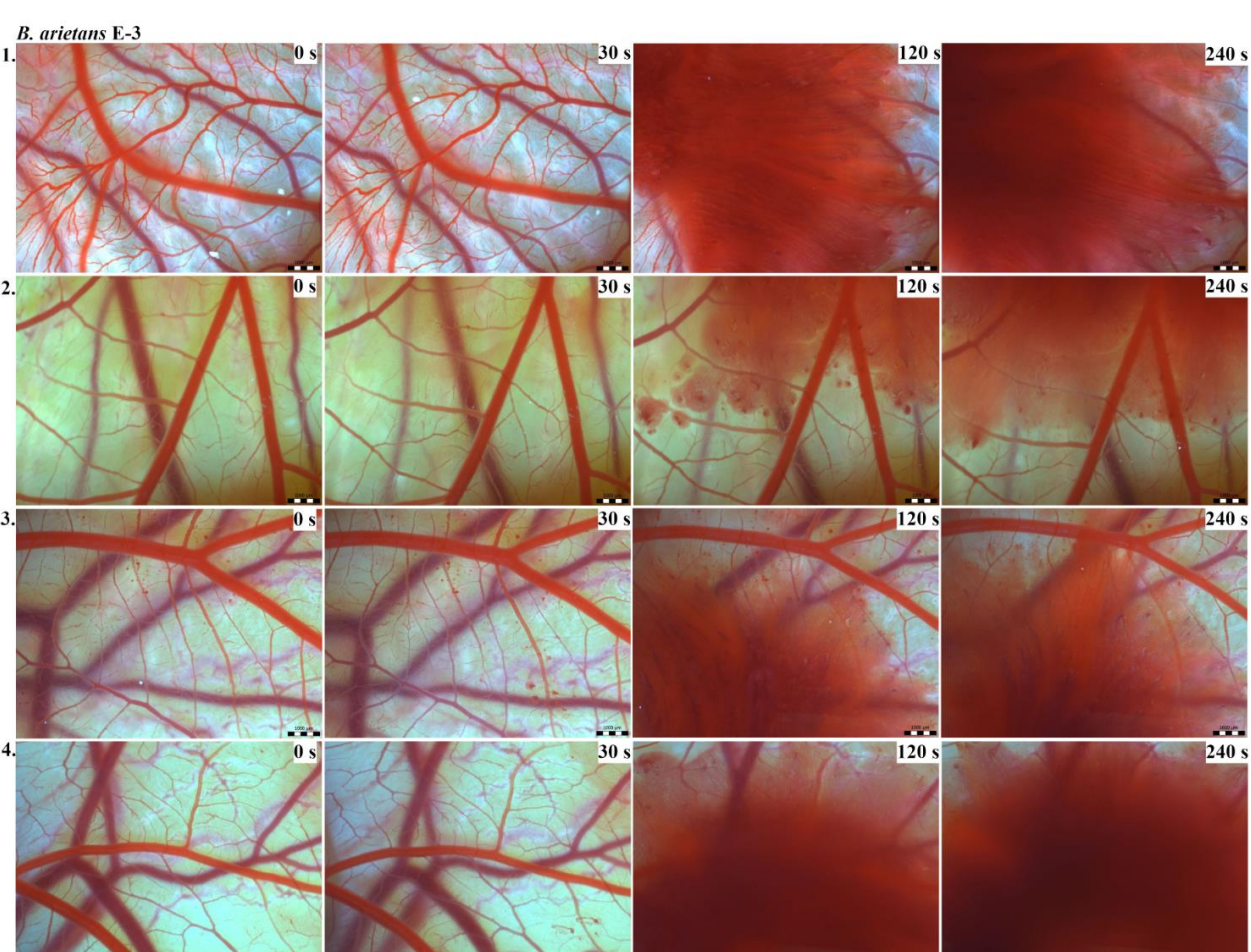
Photos of the HET-CAM test for *B. arietans* venom, concentration E-3, scale bar: 1 mm.

The results of the HET-CAM test provide a detailed insight into previously undescribed microscopic changes of CAM vessels induced by *Bitis parviocula* venom (Figure 2, Figure 3 and Figure 4). The effects observed after administration of different concentrations of *B. parviocula* venom onto the CAM surface were similar to those observed after administration of *B. arietans* venom (Figure 5, Figure 6 and Figure 7), probably due to the close phylogenetical relationship. Both venoms induced an early onset of haemotoxic effects, such as haemorrhage and formation of blood clots, within a 5-min post-application interval.

We also observed changes in vessel diameter. An interesting phenomenon was captured when vasoconstriction and vasodilatation occurred simultaneously on different parts along the same vessel, 2 min after administration of *B. parviocula* venom at the concentration of E-1 onto CAM.

According to the Luepke grading system (1985), an evaluation of scoring scheme was used to calculate the irritation potential of both assayed venoms (Table 1). From the scores obtained, we determined an average cumulative score for each concentration of each venom. Based upon the cumulative score, the irritation potential of the tested venom solutions was classified as strong, moderate and slight depending on different concentrations. We noticed a correlation between the concentration of the tested venom solution and determined irritation potential, as the average cumulative scores of both venoms decreased with reduced dilution, which is linked to the corresponding reduced dilution of the proteolytic enzymes within venoms [6]. Venoms from *B. arietans* and *B. parviocula* exhibited similar effects (haemorrhage, clotting) that varied in severity and time of onset. Venom from *B. arietans* induced rapid onset of vascular damage, bleeding, and formation of dark red blood clots within the first 30 s after application of the venom onto the CAM surface. In most cases, *B. parviocula* venom induced the first pathological effects more than 100 s after administration of the venom solution onto the CAM vessels. The highest average cumulative score was determined for *B. arietans* venom at the concentration of E-1, while the lowest average cumulative score as well as the lowest irritation potential was determined for *B. parviocula* venom at the concentration of E-3 (Table 1).

#### 2.1.2. CHEST

We recorded a higher mortality rate (Table 2) and a lower value of LD_50_ (15.48 µg/egg) after application of *B. arietans* venom, suggesting higher embryotoxic potential of venom from *B. arietans* compared to the venom from *B. parviocula* (LD_50_ = 53.53 µg/egg).

On the embryonic day 9 (9ED), significant changes in embryo weight were determined for the *B. arietans* venom, E-2 (*p* ˂ 0.01) and E-5 (*p* ˂ 0.05). We also determined significant changes in weights of embryonic hearts for the *B. parviocula* venom, E-3 (*p* ˂ 0.05) listed in Table 3.

#### 2.1.3. Acetylcholinesterase Analysis

**Figure 8:**
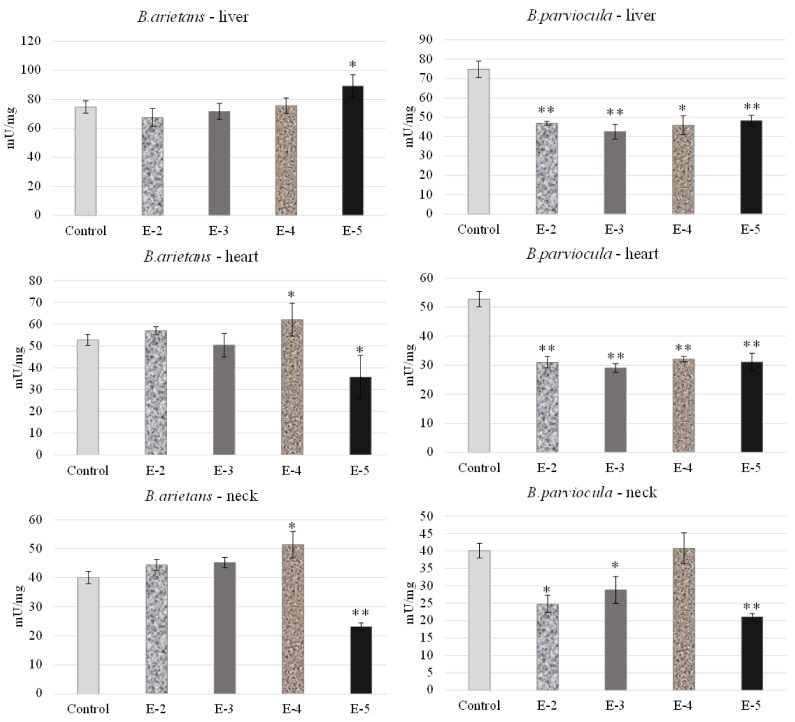
Acetylcholinesterase activity in selected organs after administration of venom obtained from *B. parviocula* and *B. arietans*. Note: Significant changes (unpaired t-test; GraphPad Prism 8.3.0. software, San Diego, CA, USA) compared to control were marked with an asterisk, where * = *p* ˂ 0.05; ** = *p* ˂ 0.01.

We observed and determined organ toxicity, expressed as the activity of acetylcholinesterase in the liver, heart and neck tissue of embryos, which were exposed to *B. arietans* and *B. parviocula* venoms. Measured results revealed an inhibitory effect of *B. arietans* venom on the livers of embryos at concentrations of E-2, E-3, E-4, but a stimulatory effect on AChE activity was observed at a concentration of E-5 (*p* < 0.05). In the heart tissue, we observed decreased AChE levels at a concentration of E-3 and E-5 (*p* < 0.05), but on the other hand, we observed a stimulatory effect at a concentration of E-4 (*p* < 0.05). Nerve tissue was obtained by homogenizing a part of the neck, where we confirmed the neurotoxic effect of the venom. Activity of AChE was reduced in the neck of embryos at a concentration of E-5 (*p* < 0.05), and significantly increased levels of AChE were observed at E-4 (*p* < 0.01). Venom from *B. parviocula* had a consistent and marked inhibitory effect at all concentrations in the livers and hearts of embryos (*p* < 0.01). Measurements in neural tissue (neck) of embryos intoxicated with *B. parviocula* venom indicated suppression of AChE activity at concentrations of E-2 (*p* < 0.05), E-3 (*p* < 0.05), and E-5 (*p* < 0.01), while increased activity of AChE was observed at E-4 (nonsignificant) (Figure 8).

### 2.2. Cytotoxicity Assays

**Figure 9:**
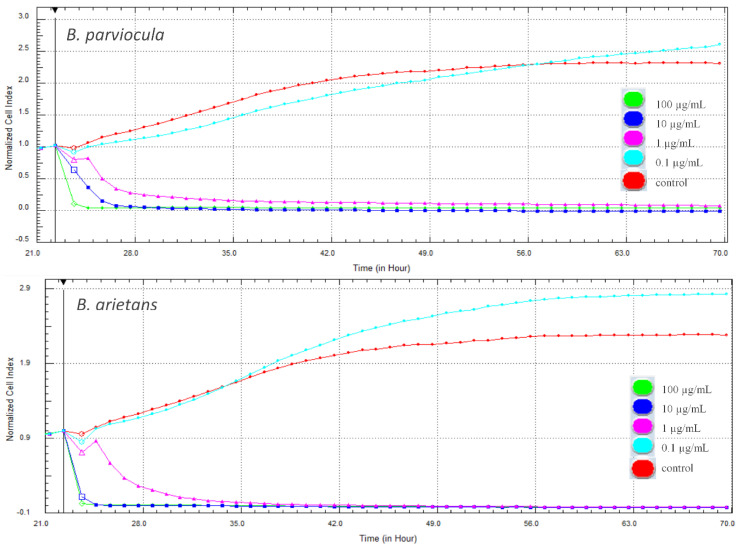
Changes in cell proliferation recorded by the xCELLigence system in real-time during treatment with tested venoms (48 h).

In our experiment, cytotoxic effects of two tested snake venoms (*B. parviocula* and *B. arietans*) on adenocarcinomic human alveolar basal epithelial cells (A549 ATCC^®^ CCL-185™) were evaluated using two different methods. The first, xCELLigence System (Real-Time Cell Analyzer—RTCA; Acea Biosciences Inc., San Diego, CA, USA) monitored the cellular response every hour throughout the duration of the experiment. Changes in proliferation and adhesion expressed in a dimensionless unit cell index (CI) were plotted in a graph (Figure 9). Cytotoxic effects of both tested venoms were evident from the first hours after their attachment to the cells, and the CI values dropped to zero. Interestingly, the lowest concentration (0.1 µg/mL) of both venoms caused a significant increase of CI when compared to control cells (*p* < 0.001).

Changes in metabolic activity were observed simultaneously with real-time cell monitoring. The cells from the same passage were treated with the tested concentrations of both venoms. After 48 h, the MTS (3-(4,5-dimethylthiazol-2-yl)-5-(3-carboxymethoxyphenyl)-2-(4-sulfophenyl)-2H-tetrazolium) assay was performed and the effect of both venoms on metabolic activity of cells was observed to be concentration-dependent. Interestingly, the same effect was observed in the following. The lowest concentration of venoms caused an increase in metabolic activity, while all the higher concentrations had a negative effect and the metabolic activity was significantly lower when compared to the control (*p* < 0.001). For better presentation, the results of changes in proliferative and metabolic activity at 48-h period of treatment were expressed as a percentage using control cells without treatment (100%). Results are presented in Table 4.

### 2.3. Snake Venom Protein Profile

**Figure 10:**
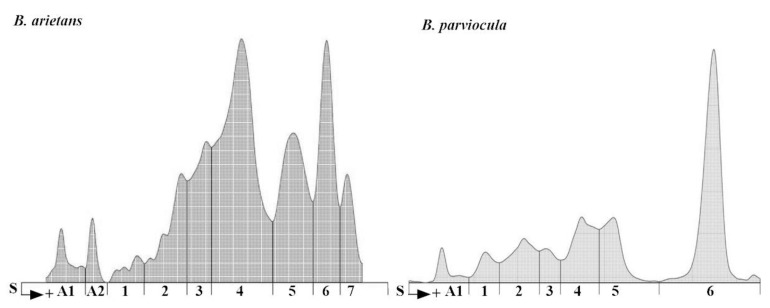
Electrophoretograms of *B. arietans* and *B. parviocula* venoms. Note: S, Start (junction between the stacking and separation gels), A1–A2, labeling of albuminlike fractions; 1–7, labeling of globulinlike fractions.

In our experiment, we analyzed protein content of the *B. arietans* and *B. parviocula* venoms. The total protein content varies from species to species (11.2 g/L versus 14.5 g/L; Table 5) as well as various separated fractions. We measured 2 albuminlike fractions in *B. arietans* venom and 1 fraction in *B. parviocula* venom. Agarose gel electrophoresis revealed 7 globulinlike fractions in *B. arietans* and 6 in *B. parviocula* venom (Figure 10). The most dominant globulinlike fraction was 4th in *B. arietans* and 6th in *B. parviocula* venom.

## 3. Discussion

The venom from the snake *B. arietans* contains components of various complexes. Disintegrins (Bitistatin, disintegrin isoforms D-2 and D-3), zinc metalloproteinase-disintegrin BA-5A, kallidin-releasing enzyme from the group of serine proteinases, post-synaptic neurotoxin Bitanarin from the group of phospholipases A2, Baptides, Hyaluronidases Hy-1 and Hy-2, True Lectins C-type CTL and CTL-2, Lectin-like C-type Proteins Snaclec, Cystatin, Vascular Endothelial Growth Factor Barietin, Fibrinogenase Ba100, and small amounts of other quantitatively under-represented components had been isolated and described in various experimental studies. Many of these components, for example, metalloproteinases and disintegrins, C-type lectins and snaclecs, serine proteinases or fibrinogenase possess the ability to affect the cardiovascular system and may contribute to haemotoxic nature of *B. arietans* venom [9,10].

In a contrast to widely studied venom from the most widespread African puff adder *B. arietans*, no information has yet been published regarding the composition of the venom from the Ethiopian endemic species *B. parviocula*. Based on similarities in vasoactive effects (haemorrhage, clotting, local hyperaemia of certain vessels) observed after administration of *B. arietans* and *B. parviocula* venom onto CAM vessels, we claim that venom from *B. parviocula* snake species may contain similar venom protein families as the venom from *B. arietans*.

CAM of chicken embryos provides a valuable model for imaging changes on vasculature and vascular injury. Both tested venoms (*B. arietans* and *B. parviocula*) showed the ability to induce blood vessel alterations on the vascular network in the chicken CAM. Haemotoxicity of *B. arietans* venom was already confirmed and documented in detail in the work published by Knight et al. (2019). Together with *B. arietans*, venoms from two other Viperid snakes (*Agkistrodon contortrix* and *Crotalus viridis*) were tested. Similarly to *B. arietans*, venoms from *A. contortrix* and *C. viridis* induced haemorrhage and clotting. No hyperaemia was observed. The irritation potential of all three venoms was very similar [6].

The mystery of the life that develops inside the chicken egg has been observed and described since ancient times. Greek philosopher Aristotle noticed significant similarities in the development of humans and chickens and summarized this knowledge in his work “*Historia animalium*”. The size of the chicken eggs allowed significant discoveries even before the invention of the microscope, and the method based on cutting a hole in the eggshell and then covering the hole with another piece of shell allowed scientists to look directly inside and observe the developing embryo [11]. Jelínek established an embryotoxicity screening test (CHEST) based on the administration of tested substances inserted onto membrana papyracea of developing embryos through a small hole in the blunt end of an eggshell, which is then covered [12]. Mortality observed 5 days after application of tested venoms allowed us to calculate LD_50_ values for both tested venoms. In toxicology, the calculation of the LD_50_ value is a method commonly used for determination of toxicity of tested substances. The mean lethal dose LD_50_ (dosis lethalis media) is the dose which, when administered at once, will cause clinical signs of intoxication in all subjects tested, and thus will cause 50% mortality among all subjects tested. After administration of the *B. parviocula* venom, only 1 dead embryo was observed among all four testing groups. This resulted in a higher LD_50_ value (53.53 µg per egg) compared to the *B. arietans* venom (LD_50_ = 15.48 µg per egg) with a total of 4 dead embryos observed in all testing groups where *B. arietans* venom was administered. In the article from 2012, the authors reported that for mice the LD_50_s of *B. parviocula* and *B. arietans* venoms were measured to be 1.56 and 1.35 mg/kg body weight, respectively [5]. Similarly, to our results, a lower LD_50_ value was determined for the *B. arietans* venom most likely as a reflection of higher toxicity.

Currently, not all components of snake venom of the genus *Bitis* are known and may vary from locality to locality. Therefore, we assume that the effect on the target organism, such as the chicken embryo in this case, will be different in our experiment. *Bitis* venom is more of a cytotoxic or hemotoxic property, but our results also suggest the presence of venom components that affect neurotransmission.

Acetylcholinesterase (AChE) is a plasma enzyme that plays a role in signal transduction in synapses and is responsible for the lysis/decomposition of acetylcholine (ACh) into choline and acetate [13,14]. Evaluation of AChE activity has many applications in the diagnosis and treatment of selected diseases [15], such as organophosphate [16] and carbamate toxicity [17], and some chemicals acting on the nervous system [18,19]. ACh participates in cell proliferation, growth, and differentiation in avians and humans [20]. During key critical periods of life, the disruptors of cholinergic functions could affect Ach activity [21].

In our experiment, we analyzed the activity of AChE in the liver of chicken embryos because AChE is synthesized in the liver. We chose the heart as an available material because the AChE-S subunit is located in muscle. Due to the fact that neurotoxic activity of selected venoms could be observed on nervous tissue, we also measured the AChE activity in the neck of embryos as the available material.

After administration of both venoms, significant AChE activity was detected in livers, hearts and nervous tissue from the neck of embryos dissected on 9ED using the CHEST assay. Our results revealed that *B. parviocula* venom had the most neurotoxic effect at all concentrations, whereas *B. arietans* venom reduced AChE activity in the liver on E-2, E-3 and in the heart, it was on E-3, E-5, while in the neck, it decreased on E-5. We discovered that the AChE activity was different in chicken embryos in most cases. Therefore, in further studies, it is necessary to analyze in detail the separated components of *Bitis* snake venom and to determine which substance can be attributed to stimulatory or inhibitory effects on the AChE activity and could have potential in the treatment of neurodegenerative diseases. We also suggest that in future experiments, the AChE activity should be monitored at different stages of embryo development (different embryonic days) and in as many organ systems as possible.

The inhibitory effect on AChE activity of some chemicals was also measured in many studies [21,22,23] observed on chick embryos. On the other hand, the stimulatory effects on AChE levels were analyzed in the study of Kalafatakis et al. [24], where high doses of the amino-acid proline increase AChE activity in target cells. Ascorbic acid [25] and rosemary acid [26] have stimulatory effects on AChE activity in the brain tissue of tested individuals.

Snake venoms have demonstrated potential anticancerous effects in model cancer cell lines and provide perspective in future drug development. The cytotoxicity of snake venom and some of its compounds (especially peptides) is known to be able to alter cellular metabolism, which is then able to act on cancer cells [27]. In our experiment, cytotoxicity of selected snake venoms on adenocarcinomic human alveolar basal epithelial cells A549 was evaluated using xCELLigence system–RTCA and the MTS test. RTCA monitors changes in cell proliferation and adhesion in real time. The loss of adherence in an adherent cell may lead to death. A strong cytotoxic effect was observed shortly after administration of the venoms to the cells, whereas CI decreased to almost zero at almost all higher concentrations (0.1–100 µg/mL). On the contrary, the lowest concentration (0.01 µg/mL) of both tested venoms caused a significant increase in CI that indicates higher adherence or cell proliferation in comparison to control.

To evaluate the results obtained by RTCA in real time, another experiment was performed on the same cell cultures using the end-point MTS assay within 48 h after administration of the venoms to the cells. We observed the dose-dependent effect, which is consistent with results obtained with RTCA. The lowest concentration of both venoms also caused an increase in metabolic activity, and higher concentrations showed a significant decrease but not as rapid as in CI. This suggests that cells are still metabolically active, but the decrease was significant when compared to the control.

Although RTCA measures many cellular activities (adhesion, spreading, proliferation), there is one important advantage over conventional end-point assays. It allows constant monitoring of cell response to substances. MTS only measures vitality and lethality at an end-point, but does not allow to predict cytotoxicity prior to this measurement [28].

Different experimental assays were carried out using specific cell lines. Cytotoxic effects of crude snake venoms were tested on cultures of malignant melanoma tumor cells. Cytotoxic substances in the venom of *B. arietans* induced apoptosis in cell lines on vascular endothelial cells of the human lung [29,30]. After administration of snake venoms (*Bitis arietans*, *Cerastes gasperettii*, *Echis coloratus*, *Echis pyramidum*, *Naja ashei*) on tumor cell lines, HCT-8 (colorectal carcinoma) and MDA-MB-231 cell lines (breast carcinoma) decreased in cell motility, colony formation and cell invasion potential. At the same time, there was an increase in oxidative stress and an increase in the production of reactive oxygen species, which led to an increased number of apoptotic cancer cells [31]. Similarly, *Naja ashei* venom induces an antiproliferative and pro-apoptotic effect in colorectal cancer cells [32].

The difference in venom composition between *B. parviocula* and *B. arietans* venoms was previously confirmed after SDS electrophoresis was performed. The authors ran 30 μg of both venoms (*B. arietans* and *B. parviocula*) on a 10–20% Tricine gel under nonreducing conditions at 150 V for 90 min using a SureXCell system. The venom from *B. arietans* contained 10 visible proteins bands, while venom from *B. parviocula* contained 13 visible bands. These results revealed quantitative differences of venom proteins, as three more proteins bands were present in *B. parviocula* venom compared to *B. arietans* venom [5].

For the purposes of our study, we performed basic protein analysis of *B. arietans* and *B. parviocula* venoms using agarose gel electrophoresis. Our results confirmed findings mentioned above, that composition of venom proteins in selected *Bitis* species are completely different. Electrophoretic separation of venom proteins was situated in human albumin and globulin regions; therefore, we used the term “albuminlike and globulinlike fractions” [33]. Similar nomenclature was employed by Göcmen et al. [33]. Total protein content was higher in the venom from *B. parviocula* (14.5 g/L) compared to the venom from *B. arietans* (11.2 g/L).

Based on the similarities of vasoactive effects observed in HET-CAM test, we suggest that the venom from *B. arietans* and *B. parviocula* may share certain venom protein families’ responsible for haemotoxicity, such as metalloproteinases and disintegrins, C-type lectins and snaclecs. Serine proteinases or fibrinogenase present in *B. arietans* venom were described to possess haemotoxic activity and may be responsible as well for similar haemotoxic effects induced by the venom of closely related *B. parviocula*. This study is the first to report that there are vasoactive compounds in the venom of *B. parviocula* with an effect on the haemoregulatory system. For further research, it is important to identify potential medically valuable compounds with haemotoxic, neurotoxic and cytotoxic properties responsible for the effects observed in our work.

## 4. Conclusions

Our results propose that *Bitis parviocula* venom contains haemotoxic components with a direct effect on vasculature of CAM and haemoregulation. In more than 90% of analyzed tissue samples (liver, heart, neck), inhibition of acetylcholinesterase was observed 5 days after the administration of *B. parviocula* venom onto the membrana papyracea of developing chicken embryos, indicating the presence of venom components that affect neurotransmission. Antitumor activity is mainly related to the antiproliferative activity of snake venoms; however, it is necessary to test the effects of individual venom compounds on both normal and tumor cells. Both tested venoms of the genus *Bitis* showed significant differences in the number and quality of fractions. We strongly recommend that, as a further step in the research, a detailed proteomic analysis should be carried out in order to identify and isolate the compounds responsible for the haemotoxic, neurotoxic and cytotoxic effects observed in our work. This might ensure the discovery of new promising agents of great medical value.

## 5. Materials and Methods

### 5.1. Snake Venom Processing

Venoms from the African viper species (*B. arietans*, *B. parviocula*) were obtained from VIPERAFARM, spol. s.r.o. (Ltd.) in accordance with cooperation agreement No. 241/2017/UVLF. The venoms were stored in a deep freezer at −80 °C. We used 170 µL of each venom. The weight of 170 µL for both venoms was 180 mg. Subsequently, the weighed volume of 170 μL of venom was diluted to a concentration of E-1, E-2, E-3, E-4 and E-5 (Table 6) [6,7].

### 5.2. Chicken Embryo Based Experimental Assays

According to legislation (2010/63/EU), the chicken embryo as an animal model did not require special approval [6]. For purposes of both experimental assays we used fertilized chicken eggs Broiler breed Ross 308 (Párovské Háje, Nitra, Slovakia). The day when cleaned eggs were placed into the incubator, pointed end downwards, was 0 embryonic day (0ED) [34]. Stable conditions (37.5 °C, 60% humidity) were maintained by automatic incubators (ET 49, ART 549) that also ensured rotation of eggs at 3-h intervals, similarly to our previous work [7].

#### 5.2.1. HET-CAM Test (The Hen´s Egg Test and Chorioallantoic Membrane)

The vascular network of chicken CAM allows imaging of changes induced by venoms [7]. The HET-CAM test was established by Luepke (1985). Vasoactive effects (hyperaemia, haemorrhage, clotting) are evaluated within 5 min post administration of a tested substance onto the CAM (Table 7). The 5-min grading interval is the most suitable for rapid effects of snake venoms [6].

From the scores obtained, the average cumulative scores for each concentration of each tested venom were determined. The gained average score correlates with the irritation potential (Table 7 and Table 8).

On embryonic day 3 (3ED) 2 mL of egg white was extracted from the pointed end of each egg, openings were sealed using molten paraffin, and the incubation continued [6].

On 9ED, administration of tested venoms´ concentrations and subsequent evaluation of vascular effects were performed. Cleaned eggs were perforated on the blunt end. The shell membrane was carefully dissected using fine forceps, exposing the chorioallantoic membrane (CAM). For every concentration of each tested venom as well as for a control, a group of 4 eggs was used, so altogether we used 28 eggs per experiment. In a control group, 50 µL of sterile saline solution was administered onto CAM. We tested different concentrations (E-1, E-2, E-3) of venoms from *B. arietans* and *B. parviocula*. We applied 50 μL of venom solution into each egg. In order to record vascular effects induced by snake venoms, photographs were taken at 0, 30, 120, and 240 s, respectively, using stereomicroscope (Olympus SZ61, Tokyo, Japan), a digital camera (Promicam 3-3CP, Prague, Czech Republic) and software (Quick Photo Micro, Promicra; Prague, Czech Republic) [6].

#### 5.2.2. Chicken Embryotoxicity Screening Test (CHEST)

A total of 90 eggs were used per experiment. On 4ED, the shell on a blunt end of each egg was perforated directly above the embryo. We administered 100 µL of tested venom solutions on the membrana papyracea. In a control group, 100 μL of sterile saline solution was applied. The openings were then sealed using transparent adhesive tape, and the incubation continued [34].

On 9ED, the eggs were opened and embryos were weighed and examined. Morphological alterations of the head, eyes, body wall and internal organs were assessed. Heart and liver were excised from each live embryo, weighed and then frozen (−80 °C) for subsequent acetylcholinesterase analysis. Changes in liver tissue might indicate compound detoxification [35].

#### 5.2.3. Acetylcholinesterase Analysis

Tissue samples (liver, heart, neck) obtained during dissection of embryos on 9ED of CHEST were frozen, stored (−80 °C) and then analyzed in order to establish the acetylcholinesterase activity induced by applied venoms obtained from the *B. arietans* and *B. parviocula* snake species.

Samples were homogenized (Sonoplus mini20, Bandelin, Berlin, Germany) in cold buffer according to manufacturer instructions. The composition of buffer was as follows: 100 mM Tris, pH 7.4, 150 mM NaCl, 1 mM EGTA, 1 mM EDTA, 1% Triton X-100, 0.5% Sodium deoxycholate, Phosphatase inhibitor cocktail, Protease inhibitor cocktail and PMSF (all Sigma-Aldrich, St. Louis, MO, USA). The tissue homogenates were analyzed using colorimetric Acetylcholinesterase Assay Kit (AChE; Abcam, Shanghai, China). The activity of AChE was recorded according to the manufacturer’s instructions and adapted for reading on microplates for an ELISA device (λ 410 nm; Multiskan^®^EX Spectrometer, Thermo-Fisher, Abingdon, UK). AChE hydrolyzed acetylthiocholine to thiocholine and acetate, and then the amount of thiocholine was quantified by DTNB (5,5′-Dithiobis(2-nitrobenzoic acid)) reagent, which was proportional to the AChE activity. The data obtained were normalized to protein values (mU/mg protein).

### 5.3. Cytotoxicity

#### 5.3.1. Cell Cultivation

Evaluation of the cytotoxic effects of the tested venoms was conducted by using adenocarcinomic human alveolar basal epithelial cells (A549 ATCC^®^ CCL-185™). The cells were cultivated at 37 °C and 5% CO_2_ with Dulbecco’s Modified Eagle Medium (DMEM) with 4.5 g L−1 glucose, L-glutamine, with 10% Foetal Bovine Serum (FBS), as well as antibiotics and antimycotics (Sigma-Aldrich, Darmstadt, Germany). Cells were passaged twice a week using cells from the same passage for the experiment, and the absence of mycoplasma contamination was checked regularly. The initial cell inoculation density was optimized by using the RTCA prior to experiments.

#### 5.3.2. Monitoring of Cell Proliferation in Real-Time

The xCELLigence (RTCA) system was used to monitor cell behavior throughout the experiment. The system monitors changes in morphology, adherence and proliferation after exposition to tested substances, and has been described in many works [36,37]. In our experiment, cells were inoculated into 16-well E-plates (Roche Applied Science, Mannheim, Germany) with density 10,000 cells/well. After initial 20-h cultivation, the cells were still within a log phase and formed an 80% monolayer. The tested substances were added to the cells at the final concentration of 0.1–100 µg/mL (E-2–E-5) calculated in triplicates and incubated for an additional 48 h. Cell changes were measured in real time each hour and recorded in a dimensionless unit cell index (CI). The more cells attached to the bottom of the well, the higher adherence, proliferation and CI activity. Proliferative activity (PA) was expressed as a percentage using control cells without treatment (100%) according to the following formula: % PA = CI_sample_ × 100/CI_control_.

#### 5.3.3. Metabolic Activity

For measurement of metabolic activity of cells, MTS colorimetric assay (CellTiter 96^®^ Aqueous One Solution Cell Proliferation Assay, Promega, Madison, WI, USA) was used. The cells were inoculated into a 96-well plate (Greiner-Bio-One, Kremsmünster, Austria) at a density of 16,500 cells per well in DMEM medium. Cells without treatment served as control (100%). After an initial incubation (20 h), tested venoms were added to the cells at the same final concentration of 0.1–100 µg/mL and incubated for another 48 h. The assay was performed according to manufacturer´s instructions and the absorbance was measured by using microplate reader (Synergy HT; Biotek, Winooski, VT, USA). Changes in metabolic activity were expressed in percentage using control cells [38].

### 5.4. Snake Venom Protein Profile

#### 5.4.1. Protein Electrophoresis

The electrophoretic determination of protein spectrum in tested venoms was performed using a Hydrasys device (Sebia, Lisses, France). Diluted venoms (E-1) were separated using a commercial kit (Hydragel 7 Proteine) in accordance with our previous study [39]. Separated protein fractions were quantified by Epson Perfection V 700 Photo densitometer scanning (λ 570 nm) and measured using Phoresis software (Version 5.50, 2009; all Sebia, Lisses, France) [39].

#### 5.4.2. Spectroscopic Determination of Venom Proteins

The total protein content of snake venoms was determined using commercial kits (Randox, Crumlin, UK). Then we measured the absorbance of the samples (λ 540 nm; Alizé device, Lisabio, Pouilly-en-Auxois, France), similarly to our previous study [39].

### 5.5. Statistical Analyses

The obtained results were assessed using GraphPad Prism 8.3.0. software (San Diego, CA, USA). For embryotoxicity and acetylcholinesterase assays, the unpaired t-test was used. All the data are summarized using means and standard deviations of relative percentage deviations and variability of standard errors within each study (SEM). The differences between groups are considered to be significant at levels of * = *p* ˂ 0.05; ** = *p* ˂ 0.01; *** = *p* ˂ 0.001. Cytotoxicity assays were performed in triplicates and analyzed using one-way analysis (ANOVA) with Dunett´s post hoc test, and the results are expressed as means ± standard deviation (SD; *n* = 3).

## Figures and Tables

**Table 1:** Average cumulative score and classification of irritation potential of *B. arietans* and *B. parviocula* venoms.

Venom	Conc.	Hyperaemia	Haemorrhage	Clotting	Total Average	Irritation Potential
Control	0	0	0	0	0	0
*B. arietans*	E-1	0	7	9	16	Strong
	E-2	0	6	5.5	11.5	Strong
	E-3	0	6	0	6	Moderate
*B. parviocula*	E-1	0	6	7	13	Strong
	E-2	0	5	1.25	6.25	Moderate
	E-3	0	4.5	0	4.5	Slight

Note: Conc. = concentration.

**Table 2:** The results of the chicken embryotoxicity screening test (CHEST) test for each dilution of *B. arietans* and *B. parviocula* venoms.

Snake Venom	Conc.	N	Live Embryos	Dead Embryos	Mortality(%)
Control	0	10	10	0	0
*B. arietans*	E-2	9	6	3	33.33
	E-3	8	7	1	12.5
	E-4	9	9	0	0
	E-5	10	10	0	0
*B. parviocula*	E-2	10	9	1	10
	E-3	10	10	0	0
	E-4	10	10	0	0
	E-5	10	10	0	0
Total		86	81	5	

Note: Conc. = concentration, N = number of chicken embryos.

**Table 3:** Weight changes (g) during CHEST test after application of selected venoms (*B. arietans* and *B. parviocula*).

	Venom Dilution	Control Group	B. arietans	B. parviocula
Body weight	0	0.9185 ± 0.0341		
	E-2		0.7225 ± 0.0236 **	0.8529 ± 0.053
	E-3		0.8769 ± 0.0422	0.8541 ± 0.0382
	E-4		0.8443 ± 0.0523	0.8561 ± 0.0273
	E-5		0.8119 ± 0.032 *	0.9597 ± 0.0211
Heart	0	0.0077 ± 0.0008		
	E-2		0.0062 ± 0.0011	0.0071 ± 0.0008
	E-3		0.0073 ± 0.0003	0.0055 ± 0.0005 *
	E-4		0.0067 ± 0.001	0.0073 ± 0.0005
	E-5		0.0064 ± 0.0004	0.0082 ± 0.0007
Liver	0	0.009 ± 0.0008		
	E-2		0.0085 ± 0.0016	0.009 ± 0.0012
	E-3		0.0117 ± 0.0017	0.0081 ± 0.0009
	E-4		0.0099 ± 0.001	0.0097 ± 0.0011
	E-5		0.0076 ± 0.0005	0.011 ± 0.0009

Note: Significant changes compared to control were marked with asterisk, where * = *p* ˂ 0.05; ** = *p* ˂ 0.01.

**Table 4:** The effect of tested substances on proliferation and metabolic activity after 48 h (%).

		PA (%)	±SD	MA (%)	±SD
*B. arietans*	E-5	124.20	±3.3 ***	122.05	±6.3 ***
	E-4	0.77	±0.01 ***	73.89	±1.72 ***
	E-3	1.16	±0.01 ***	28.17	±0.63 ***
	E-2	0.75	±0.01 ***	24.42	±0.22 ***
*B. parviocula*	E-5	112.63	±2.1 ***	94.15	±5.83 ***
	E-4	3.18	±0.05 ***	53.60	±3.35 ***
	E-3	0.71	±0.02 ***	31.65	±0.4 ***
	E-2	1.6	±0.07 ***	23.7	±0.16 ***

Note: PA—cell proliferation; MA—metabolic activity of the cells. Significant changes compared to control were marked with an asterisk, where *** = *p* ˂ 0.001.

**Table 5:** Protein fractions and total protein content in *B. arietans* and *B. parviocula* venoms.

		*B. arietans*		*B. parviocula*	
Name of Fraction		%	g/L	%	g/L
Albumin-Like Fractions	A1	2.8	0.3	3.8	0.6
	A2	2.1	0.2	no	no
Globulin-Like Fractions	1	2	0.2	5.6	0.8
	2	9.2	1.1	11.8	1.7
	3	11	1.2	5.6	0.8
	4	34.5	3.9	17	2.5
	5	17.2	1.9	13.6	2
	6	15.6	1.7	42.6	6.2
	7	5.6	0.7	no	no
Total Proteins		100	11.2	100	14.5

**Table 6:** Concentrations of tested snake venoms solutions.

	Concentration µg/µL					
Snake Venom	Origin	E-1	E-2	E-3	E-4	E-5
*B. arietans*	South Africa	106	10.6	1.06	0.106	0.0106
*B. parviocula*	Ethiopia	106	10.6	1.06	0.106	0.0106

**Table 7:** Scoring scheme for irritation potential calculation according to Luepke (1985) applied to the HET-CAM method.

	Score		
Time Taken for Manifestation of Irritation Effect	Hyperaemia	Haemorrhage	Clotting
<0.5 min	5	7	9
0.5–2 min	3	5	7
2–5 min	1	3	5

**Table 8:** Classification of irritation potential based upon cumulative score according to Luepke (1985).

Cumulative Score	Irritation Potential
<1.0	Negligible
1.0–4.9	Slight
5.0–8.9	Moderate
9.0–21.0	Strong

## Data Availability

Additional data are available on request from the corresponding author.
